# Spatially intermixed objects of different categories are parsed automatically

**DOI:** 10.1038/s41598-020-79828-4

**Published:** 2021-01-11

**Authors:** Vladislav A. Khvostov, Anton O. Lukashevich, Igor S. Utochkin

**Affiliations:** Psychology Department, HSE University, Armyansky per., 4, building 2, Office 419, Moscow, Russian Federation 101000

**Keywords:** Psychology, Neuroscience, Cognitive neuroscience, Visual system

## Abstract

Our visual system is able to separate spatially intermixed objects into different categorical groups (e.g., berries and leaves) using the shape of feature distribution: Determining whether all objects belong to one or several categories depends on whether the distribution has one or several peaks. Despite the apparent ease of rapid categorization, it is a very computationally demanding task, given severely limited “bottlenecks” of attention and working memory capable of processing only a few objects at a time. Here, we tested whether this rapid categorical parsing is automatic or requires attention. We used the visual mismatch negativity (vMMN) ERP component known as a marker of automatic sensory discrimination. 20 volunteers (16 female, mean age—22.7) participated in our study. Loading participants’ attention with a central task, we observed a substantial vMMN response to unattended background changes of categories defined by certain length-orientation conjunctions. Importantly, this occurred in conditions where the distributions of these features had several peaks and, hence, supported categorical separation. These results suggest that spatially intermixed objects are parsed into distinct categories automatically and give new insight into how the visual system can bypass the severe processing restrictions and form rich perceptual experience.

## Introduction

Every moment, our visual system deals with many objects in a visual scene. Despite the severe restrictions of attentional and working memory capacities^1,2^ precluding deep simultaneous processing of all objects^3^ the visual system has much more information than these limits predict. One possible solution to this contradiction is the idea that the visual system extracts *ensemble summary statistics* from the whole set without holding information about each individual item^4^. It was shown that observers can extract mean^5–8^ and variance/range^9,10^ of some features for a set of objects. Also, observers can rather accurately estimate an approximate number of objects^11,12^ (however the debate whether it is an independent ability is still ongoing^13^). The broad spectrum of features can be compressed into ensemble statistics: size^5^, orientation^9^, emotional expression^14^, animacy^15^, etc. Ensemble summaries can be represented perceptually rather than inferred which is supported by evidence from adaptation aftereffects^11,16^. Ensemble information is extracted rapidly (as quickly as 50–200 ms^6,17^) and often with no or limited conscious access to individuals^5,8,18^. Recent studies showed that the visual system can represent the whole distribution of features^19^. It suggests that mean, variance, and numerosity are not the only things representing ensemble information. Rather, these studies suggest that ensemble representations store quite rich information about the whole set of objects that can be useful for many cognitive tasks.

One possible application of knowing the whole distribution is rapid categorization. In everyday perception, we often deal with sets of spatially interleaved objects of different types. A typical example is berries and leaves on a bush. Here, it makes more sense to split the berries and the leaves into independent groups before calculating ensemble summaries across these two sets. Indeed, this is what the visual system does. Not all objects necessarily get compressed into a single ensemble percept, one for all. The visual system can easily and rapidly parse a lot of interleaved objects into different color subsets and independently calculate ensemble summaries for each group separately^7,20,21^. Orientation can also serve as a cue for parsing into subsets, though not ideally^22,23^. To be capable of such independent computations, the visual system has to rapidly “decide” that some elements are similar enough to include them in the same pool to process as an ensemble, whereas others are substantially different to exclude them from that pool. This “decision” that we term *rapid ensemble-based categorization* or *segmentation* requires access to more elaborated distributional properties than the grand mean or variance of the entire set.

It was previously suggested that the ensemble-based segmentation of spatially overlapping subsets can be supported by the shape of an overall feature distribution along one or several visual dimensions^24^. If the distribution has a single peak or is relatively flat (*non-segmentable* distribution), the visual system would treat all objects as belonging to one category. In contrast, if the distribution has several peaks and long gaps between them (*segmentable* distribution), this would more likely cause the perception of a set consisting of objects from different categories. In our example, the visual system treats berries and leaves as different types of objects because their features (e.g., colors, shape) are distributed in a bumpy manner. But we will likely see a single type of objects looking at autumn leaves on the ground because their color distribution contains many intermediate shades between red and green forming a flat, single-peak distribution. Empirical evidence for this theory comes from visual search^25^ and texture discrimination^26^.

In light of the idea of the efficient ensemble representation beyond the bottleneck of attention and working memory^27^, it is a debated question whether ensemble processing in general and ensemble-based categorization in particular require no attention: While some behavioral studies suggest efficient ensemble perception when attention is occupied by another task^8,22^ other studies show that at least some distributed attention is required for ensemble processing^28,29^ or, at most, the whole ensemble percept is heavily based on attentional subsampling of a few items^30^. Ensemble-based segmentation occurs rather early (within 100–200-ms) and does not benefit from longer presentation^26^ which is consistent with presumably parallel processing associated with automatic, “preattentive” segmentation or categorization. However, the conclusion about preattentive versus attention-demanding ensemble segmentation based solely on behavioral data is problematic because such tasks explicitly require discrimination based on ensemble properties implying that these properties are attended. In this work, we addressed this problem and tried to figure out whether the rapid segmentation of intermixed objects is automatic. Specifically, our approach was based on probing a neurophysiological correlate of automatic discrimination, visual mismatch negativity (vMMN).

The MMN component of the ERP is known as a correlate of automatic change detection in the sensory input^31^. The visual system is capable of generating a mismatch signal while responding to the violations in unattended environmental regularities^32^, both in physical parameters such as color or orientation^33^ and in high-level features including facial expression^34^ or even in abstract rules^35^. The common method to probe the vMMN is the oddball paradigm, with a central task diverting participant’s attention and a background stimulus stream consisting of alternation between standard (frequent) and deviant (rare) stimuli. The typical vMMN is greater negative activity in response to the deviant compared to standard stimulus taking place in a 120–250 ms time window and topographically distributed in occipital and parietal electrode sites. To our knowledge, approaches to the control of attention vary across the field of vMMN research and include demanding central tasks^36^, attentional blink^37^, and even no concurrent attentional task^38^. Yet, vMMN as a posterior negativity has been consistently found regardless of these manipulations. Recent research on the effect of task difficulty on the vMMN showed various, sometimes contradictory results^39,40^. It appears to be important to keep balance in task difficulty to divert attention and prevent the participants exhaustion. We designed our paradigm so that central changes were independent of background texture changes, which is an effective way of stimulus organization to dissociate the effects of the two types of changes^38^. Therefore, our method of manipulating attention is in line with existing approaches in the vMMN literature.

To answer the question about the automaticity of rapid categorization using vMNN, we presented participants textures filled with lines differing in length and orientation while participants’ attention was occupied by another task. The distributions of lengths and orientations could be either two-peaks (segmentable) or uniform (non-segmentable). A deviant event was the change of a length-orientation correlation sign (e.g., standard textures contained lines following “the longer–the steeper” rule, whereas deviant textures contained “the longer–the flatter” lines). Importantly, standard and deviant textures had identical distributions of lengths and orientations, so that they could not be discriminated based on simple summary statistics such as mean or variance. Rather, the discrimination could be based only on more localized subset analysis which necessarily implies rapid categorical parsing: e.g., detecting that ‘categories’ of long-steep and short-flat lines in standard stimuli are replaced by ‘categories’ of long-flat and short-steep lines in deviant stimuli. The results of the previous behavioral study with similar stimulation^23^ predict that such discrimination will occur only for textures consisting solely of highly distinct, segmentable features that can provide clear, non-confusable categories. Thus, the vMMN (if present) in our paradigm would most likely reflect an ability to automatically detect the change in the statistics of multiple intermixed objects parsed into separate categories.

## Method

### Participants

Minimum a priori sample size was set at fifteen participants, based on sample sizes typical for many recent vMMN studies using similar designs^33,41^. To meet potential technical problems, we recorded ERP data from 20 (16 female, mean age—22.7 years) neurologically typical students who participated as volunteers. All participants had normal or corrected-to-normal vision and gave written informed consent. The protocol was approved by the Research Ethics Committee of the Psychology Department, Higher School of Economics and followed the Declaration of Helsinki guidelines.

### Stimuli and procedure

**Figure 1:**
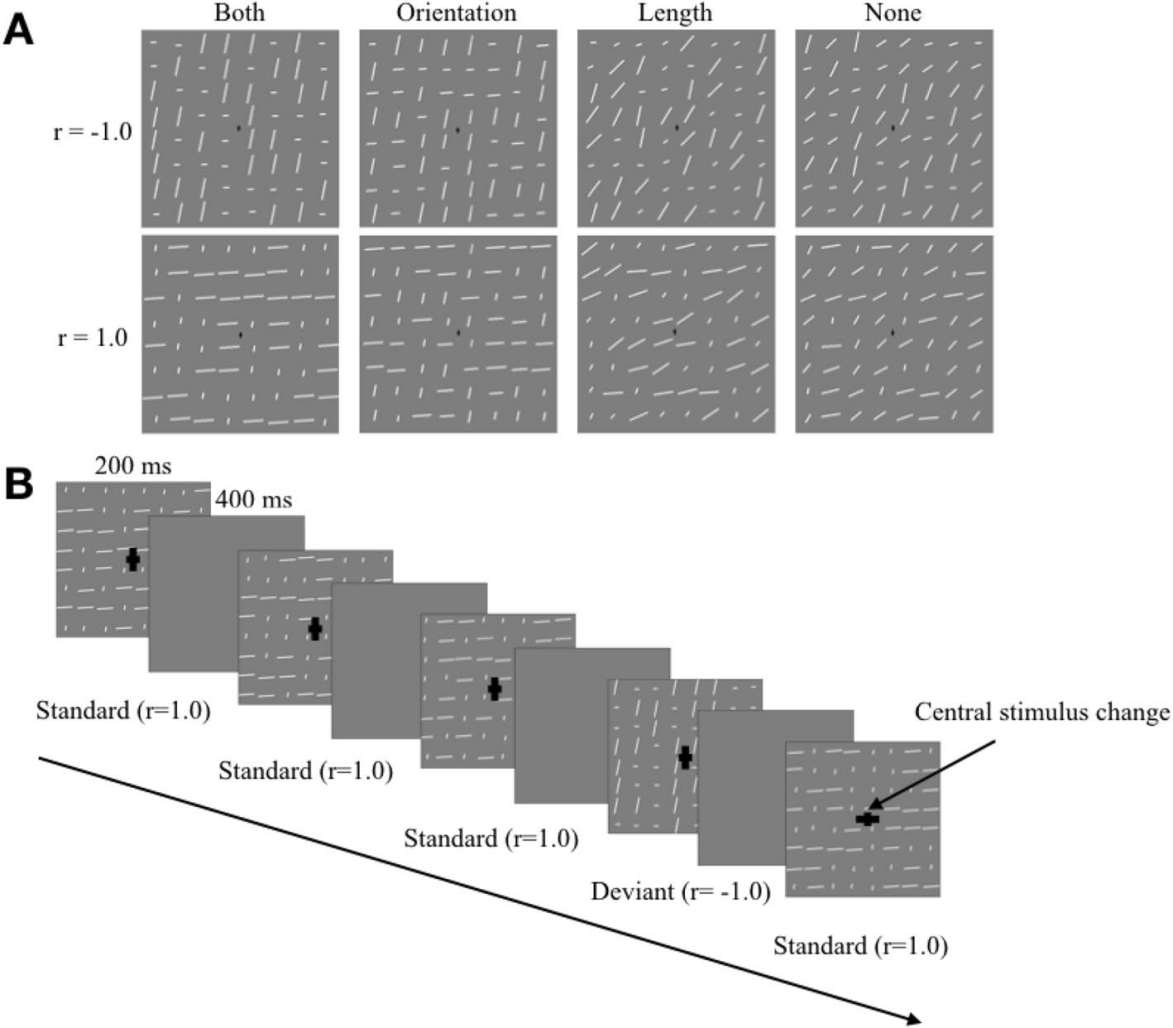
Methods. (**A**) Example stimuli used in the four segmentability conditions of the experiment with two possible length-orientation correlations (*r*’s). (**B**) The time course of a typical trial in the experiment (“both” condition). Participants were instructed to press the “D” button whenever the central cross changed its orientation. In the oddball trials, the background texture changed the length-orientation correlation to the opposite. The central cross size is magnified for illustrational purposes.

The experiment was run using Presentation software (Version 18.0, Neurobehavioral Systems, Inc., Berkeley, CA, www.neurobs.com). Participants sat 90 cm from the monitor: one pixel was equal to 0.02° of visual angle from this distance. To provide textures for which the MMN were recorded, we generated sets of 64 white lines randomly fit in an 8 × 8-cell square grid subtending 8.62° × 8.62° area. All lines had a constant width of 0.06° and varied in length and orientation. Lengths varied between 0.33° and 1.11° with an increment step of 0.05° yielding 16 unique length values. Orientation varied between 11° and 86° with steps of 5° (also 16 unique orientation values). A small black cross (0.45 cd/m^2^) with either longer vertical or longer horizontal hand (0.33° and 0.16°) was placed in the center of the texture and used both for gaze fixation and for the central attention-engaging central task. The distributions of lengths and orientations among textural elements (lines) were manipulated in terms of their shape to provide different degrees of “segmentability”. In each of the dimensions, the distribution could be either “segmentable” (two-peaks distribution consisting of only extreme values presented in equal proportions) or “non-segmentable” (uniform distribution consisting of extremes and all transition steps in equal proportions). Given the orthogonal manipulation of segmentability in length and orientation, we had four segmentability conditions: “both” (length and orientation are segmentable), “orientation” (only orientation is segmentable), “length” (only length is segmentable), and “none” (none of the features are segmentable). Figure 1A illustrates these four conditions. Importantly, lengths and orientations were strictly correlated within each display but the sign of this correlation was different across displays. If a correlation was* r* = − 1 then the longer the line, the steeper it was (Fig. 1A, top row). If a correlation was *r* = 1 then the longer the line, the flatter it was (Fig. 1A, bottom row). All displays differed from each other in the spatial distribution of elements, so that no individual length-orientation conjunction repeated at the same position many times in a row. Therefore, the sign of correlation was the only determinant of statistical differences between textures within each segmentability condition, whereas the feature distributions stayed constant and individual elements randomly changed their locations.

The experiment had a within-subject blocked design. Each block consisted of a series of standard and deviant stimuli belonging to the same segmentability condition and differing only in the sign of length-orientation correlation. Each stimulus was presented for 200 ms followed by a non-jittered interstimulus interval of 400 ms. Participants were instructed to fixate a central cross and track its changes over time (central oddball task). They had to press a “D” button on a keyboard whenever the cross changed its orientation from vertical to horizontal (Fig. 1B). This central task was used to divert attention from textures. Central changes could occur only in standard trials. Oddball events for the cross and for the background textures were assigned to random, uncorrelated temporal positions in a block.

Each participant was exposed to eight blocks of trials (4 segmentability conditions × 2 length-orientation correlations in a deviant vs. standard stimulus). The order of blocks was randomized across participants. The probabilities of the central and background oddball events in each block were 7.5% and 10% respectively (note, these two events could not happen in the same trial). The overall number of trials per block was 700 (630 standard and 70 deviant stimuli).

### EEG recording

EEG was recorded using the ActiCHamp amplifier with 64-channel active AgCl electrodes (actiCHamp Plus, Brain Products GmbH, Gilching, Germany) placed on the scalp according to the modified 10–20 system. Both mastoid electrodes were used as reference. A ground electrode was placed on the participant’s forehead. The horizontal electrooculogram was recorded with a bipolar configuration between electrodes positioned lateral to the outer canthi of the two eyes. Vertical eye movements were monitored with a bipolar montage between electrodes placed above and below the right eye. Recording was performed with an on-line 50 Hz notch filter. After recording, the data was resampled to 500 Hz rate and re-referenced to the grand average. Off-line filters with a high cut-off at 0.1 Hz and low cut-off at 35 Hz were applied. EEG preprocessing was performed using BrainVisionAnalyzer software (BrainVision Analyzer, Brain Products GmbH, Gilching, Germany**).**

Ocular artifacts were rejected using the ocular correction ICA algorithm.

### Data analysis

Behavioral data was analyzed in terms of the central task accuracy. We calculated the percentage of correct answers for reporting the change in the central cross. Both misses (a participant did not press the button, but the cross was changed) and false alarms (a participant pressed the button, but the cross was not changed) were taken into account as errors.

**Figure 2:**
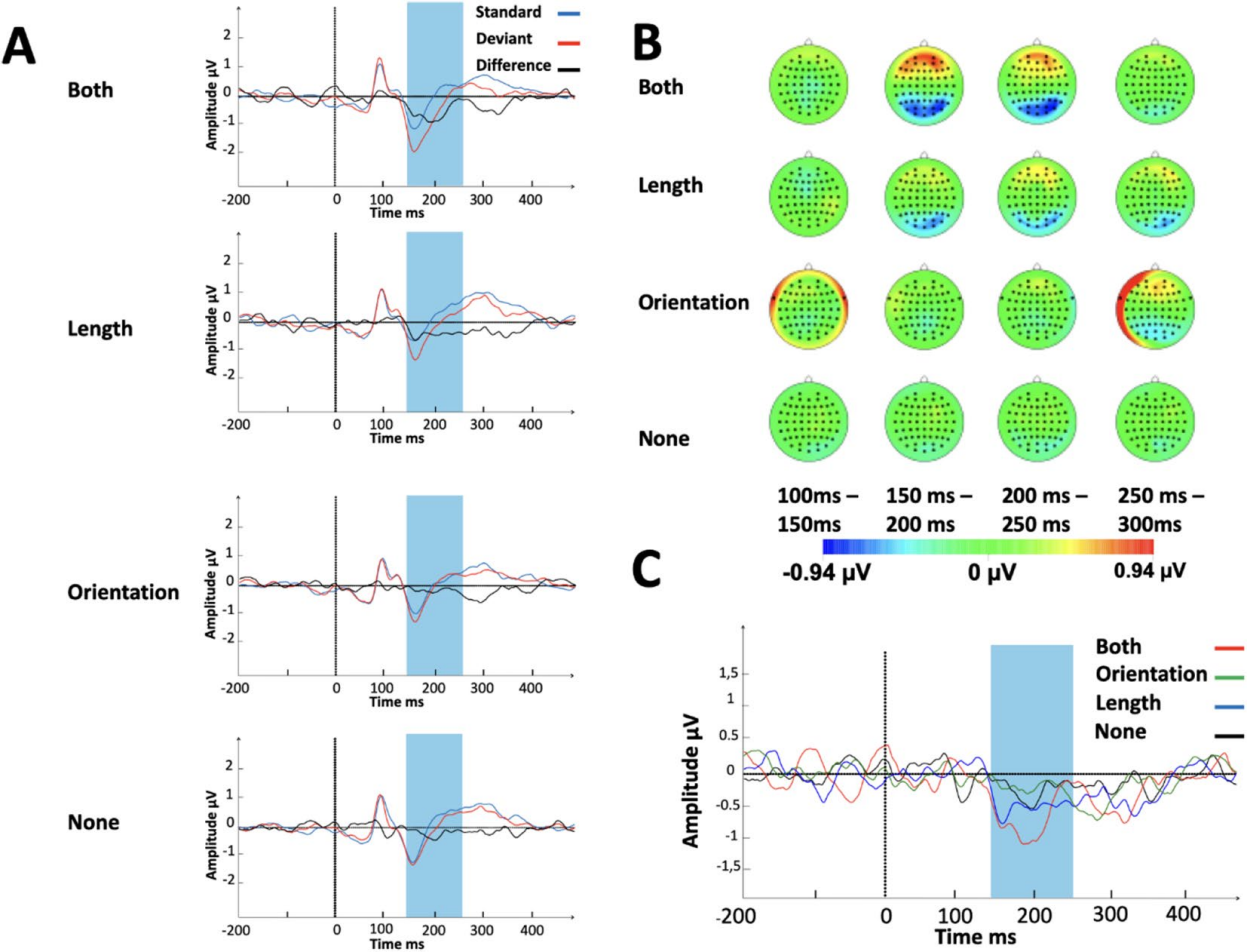
Electrophysiological results of the experiment: (**A**) grand average waveforms with difference waves in posterior electrode sites in each of the segmentability conditions. (**B**) The scalp distribution of the event-related potential in different segmentability conditions split by temporal windows. (**C**) Grand averages of the difference waves as a function of the stimulus condition. Created using: BrainVision Analyzer, Brain Products GmbH, Gilching, Germany.

For ERP-analysis, we extracted 700-ms length epochs including 200 ms of the pre-stimulus baseline period. The baseline was corrected among all segments. Trials with central change were excluded from the analysis. The epochs were averaged separately for the standard and deviant trials within each segmentability condition regardless of the sign of length-orientation correlation. We aimed to average 140 randomly predefined standard and 140 deviant epochs per condition uniformly distributed across each block. After preprocessing procedures, the average epochs number per condition per participant was 130 (SD = 11.9). To avoid the problem of averaging the amplitudes between positive and negative parts of the ERP curve, we obtained the difference wave by subtracting the response to standard from the response to deviant stimulus (Fig. 2A). Visual inspection of topographical scalp potential distribution of difference waves showed a negativity within 100–400 ms time window in following electrode sites over posterior region: O1, Oz, O2, P1, Pz, P2, P3, P4, P5, P6, P7, P8, POz, PO3, PO4, PO7, PO8 (Fig. 2B). Difference waves in these sites were combined and used for all analyses below (Fig. 2C).

To specify the precise time window for the statistical analysis of the vMMN, we used a series of point-by-point one-sample *t*-tests (left tailed), comparing the amplitudes of the difference wave’ in the four segmentability conditions against zero within the 100–400 ms time interval^42^. Only significant negative deviations in at least 12 consecutive data points (24 ms) were considered to indicate the presence of the vMMN. The corresponding time points were included in the final time window. After determining the time window, we calculated the mean amplitude of the difference wave over this time period and compared it against zero to determine the presence of the vMMN in each segmentability condition. We also ran a one-way repeated-measures ANOVA for these values to compare the vMMN differences between the conditions.

The statistical analysis was run using standard significance tests and Bayes factors. In the Bayesian statistical inference, the Bayes factor (BF_10_) is the odds showing the relative likelihood of the H_1_ against the H_0_ given the data. The Bayes factors were calculated in JASP statistical software (JASP 0.11.0.1; JASP, Amsterdam, the Netherlands). Jeffreys’s scale^43^, with Kass and Raftery’s adjustment^44^, was used to interpret the Bayes factors.

## Results

Data of five participants were excluded from analysis due to the prevalence of alpha rhythm and technical issues. Therefore, the data from fifteen participants were analyzed. All data including raw EEG can be found at https://osf.io/tymv7/.

### Behavioral data

The percentages of correct answers in the central task were very high (> 92%) in all segmentability conditions. Presumably, most of the errors were caused by a short time window to respond (i.e., a participant noticed a central change but pressed the button too late, when the next trial has already started). This led to a recognizable pattern in the data: a trial with the change is marked as a miss and a next trial is marked as a false alarm. Overall, we found no effect of segmentability on the error rate (*F*(3,42) = 1.859, *p* = 0.145, *η*_p_^2^ = 0.119*, BF*_10_ = 0.564). Mauchly’s test indicated that the assumption of sphericity had not been violated (*χ*^2^ = 6.81, *p* = 0.236). From the visual search literature, we know that the low frequency of a target event (central oddball change in our case) makes the detection task harder^45^. Therefore, based on such good performance rates, we conclude that our participants were attentionally engaged in the central task in all texture segmentability conditions. This lets us conclude that observers’ attention was mostly diverted from the background textures, which is important for the interpretation of the MMN.

### Electrophysiological data

Using the criterion described in Data analysis section (12 consecutive data points of significant negative deviation from zero), we revealed a reliable early negative deviation from the baseline within a 150–236 ms time window for the “both” condition, within 154–266 ms—for the “length” condition, and within 188–220—for the “none” condition. These deviations in the “both” and the “length” conditions had earlier latencies than that in the “none” condition. For the “orientation” condition, we found no reliable deviation from the baseline. Based on this, we defined our time window of interest as 150–266 ms to grasp a potential vMMN in all conditions at early stages of visual processing. In addition, we discovered the presence of a second negative “component” in some of the conditions, namely, within 294–360 ms for the “both” condition, 318–362 ms for the “length” condition, and 268–314 ms—for the “orientation” condition. However, such components with latency more than 300 ms likely reflect processes involving attention to some degree^46^. We did not include these latencies in our time window of interest because our primary focus is on early, preattentive, automatic processing of stimuli.

**Figure 3:**
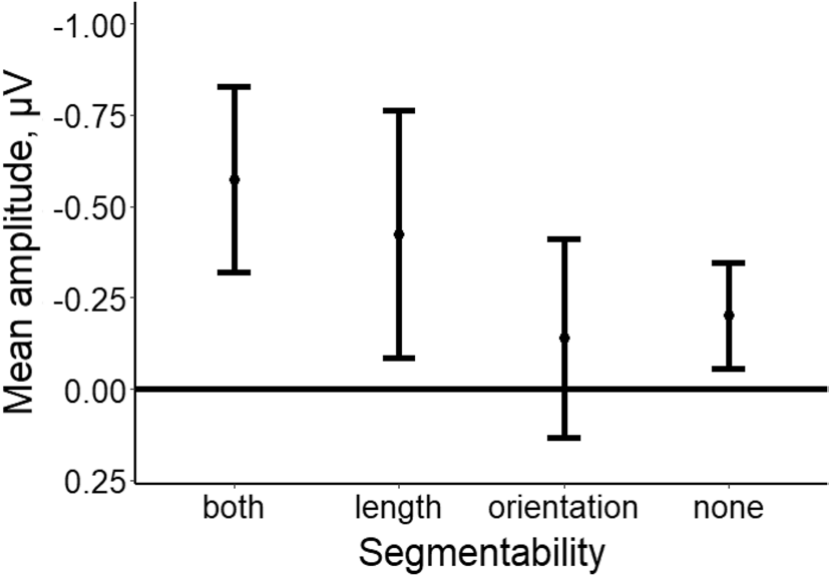
The mean amplitude of the difference wave within a 150–266 ms time window as a function of segmentability. Error bars denote 95% confidence intervals, with between-subject variance removed in accordance with the Cousineau method^47^.

Mean amplitudes of difference waves were calculated for the time window of 150–266 ms (Fig. 3). Direct comparisons against zero (left tailed t-test) showed evidence of the presence of the vMMN for the “both” (*t*(14) = 3.572, *p* = 0*.*002*,* Bonferroni corrected *α* = 0.012, *d*_z_ = 0.922, *BF*_10_ = 29.768) and the “length” (*t*(14) = 2.833, *p* = 0*.*007*, d*_z_ = 0.732, *BF*_10_ = 8.741) conditions. For the “none” condition, we obtained borderline results (*t*(14) = 2.538, *p* = 0*.*012*,* Bonferroni corrected *α* = 0.012, *d*_z_ = 0.655, *BF*_10_ = 5.45) and the “orientation” condition showed no evidence for the vMMN (*t*(14) = 1.011, *p* = 0*.*165, *d*_z_ = 0.216, *BF*_10_ = 0.667). Repeated-measures ANOVA showed no effect of segmentability on the mean amplitude of difference waves (*F*(1.763, 24.681) = 2.035*, p* = 0.156*, η*_p_^2^ = 0.127*, BF*_10_ = 0.95). Note that Mauchly’s tests indicated the violation of the assumption of sphericity (χ^2^(5) = 15.849, *p* = 0.007) so degrees of freedom, F-statistic, and *p* value were corrected using Greenhouse–Geisser correction.

The present results show that electrophysiological activity in posterior regions in response to deviant stimuli was greater than in response to standard stimuli (within early 150–266 ms time window) for “both” and “length” conditions which indicates the presence of the vMMN. At the same time, the other two segmentability conditions did not show reliable difference between standard and deviant stimuli. Therefore, we conclude that there was no strong evidence for vMMN in these conditions.

## Discussion

In this study, we used the vMNN as an indicator of automatic sensory processing for testing whether the categorical parsing of multiple objects based on statistical distributions of their features is automatic. Our main result was the finding of the early vMMN in the “both” and the “length” conditions when both feature distributions or at least length were segmentable. In contrast, there was no strong evidence for the vMMN in the other two conditions. Given our sample and the low-level character of the task, we consider our results broadly generalizable to the population of neurotypical observers.

As a general case, global texture discrimination based on the correlation between the features when the feature statistics are kept fixed across the textures is a difficult task^48,49^. Some of the prominent theories suggest that global preattentive processing is only capable of detecting large differences in simple feature statistics^48,50^ and that focal attentional processing is required to process more complex feature conjunctions. Our vMMN results demonstrated that this is not always the case. We kept the distributions of lengths and orientations the same, so no simple feature statistics could be used to discriminate between standard and deviant textures. Therefore, our correlation manipulations could be globally detected only as conjunction-based differences. Contrary to the predictions of the aforementioned theories, the finding of the vMMN in such a task indicates that there can be some early discrimination of length-orientation correlations that occurs when focused attention is engaged with another task.

However, the MMN in our study was modulated by the shape of the feature distributions, as considerable MMN were observed only when the feature distributions had clear peaks. We interpret this finding in terms of rapid segmentation and categorization. As proposed earlier^24^, the two-peaks distributions support good segmentability. We suggest that when length and orientation distributions were two-peaks (“both” condition), they supported the segmentation into categorical subsets that could be then contrasted across textures (e.g., long-steep vs. long-flat). Importantly, this vMMN result matches the previously reported behavioral pattern^26^ showing that participants could perform an explicit texture discrimination task with similar stimuli only when both features had a two-peaks distribution. Given this resemblance between the occurrence of the vMMN and the rise of texture discrimination in the behavioral experiments, we conclude that an early automatic process can contribute to the rapid segmentation of categorically distinct sets of objects based on their ensemble statistics. This is also in line with a finding that the segmentability effect on discrimination quickly grows within 200 ms and stays approximately the same at later durations, that is, it does not benefit from the serial deployment of attention (cf.^51^). Unlike behavioral results, where the segmentability effect was found only for the condition with both segmentable distributions, the current study also showed the vMMN in the “length” condition where only one distribution was segmentable. At the same time, similar vMMN was not found in the condition where another feature, orientation was segmentable alone. One possible explanation is that feature separation in the segmentable length distribution was a stronger supporter of preattentive segmentation whereas orientation separation alone was insufficient. A more sophisticated explanation can come from the differences in the nature of length and orientation as feature dimensions. Length, or size in general is an asymmetrical sensory dimension in a sense that bigger elements are usually more salient among small ones than vice versa^48,52^. Therefore, if long lines were well segmented, they could further automatically bias orientation comparison toward a category of long lines (picking long lines and detecting a change in their mean orientation). Presumably, this did not occur when only orientations were segmentable because orientation is not an asymmetrical feature dimension and, thus, would not bias processing to the steep or flat category based on automatically detected saliency. These suggested explanations need thorough testing in the future research.

To recapitulate, our analysis of rapid ensemble-based categorization started with an example of seeing berries among leaves on a bush. Our results show that, if a subset of items is distinct (segmentable) from another subset then these subsets are differentiated automatically from each other. Previous work on the perception of spatially non-overlapping textures has shown that they can be segregated effortlessly and automatically if supported by substantial differences in region statistics^53–56^. For such spatial organization, the ease of segmentation can be explained by known properties of low-level visual organization, with retinotopic structure and local interactions, such as lateral inhibition^57^. Here, we provide evidence that even in poor spatial organization, spatially intermixed items of different kinds still can be automatically determined as belonging to different categories. Ensemble representation of the overall feature distribution can be a potential basis for such categorization. It is important to note that spatial overlap in some cases interferes with individual subset processing, even if the subsets are perfectly segmentable^20,22,23,26^. This may indicate some additional difficulties with the suppression of irrelevant subsets and, hence, suggests the role of attention in multi-location selection. However, these selection and suppression issues appear not to influence categorization itself that, according to our data, can occur “preattentively”, that is, prior to the selection stage.

In this study, we tested whether the visual system can automatically detect the violation of the statistical structure of a texture. Although texture differences were defined in terms of length-orientation correlation, we presume that our paradigm was aimed to test mostly rapid ensemble-based categorization rather than correlation perception per se. In support for this claim, our previous behavioral data^26^ showed that, as a general case, people are practically insensitive to even extreme correlation changes: Observers had extremely low sensitivity (*d*′ = 0.0–0.3) even when two texture patches had correlations *r* = 1 and − 1. However, observers were substantially better at texture discrimination (*d*′ = 0.7–0.8) when both length and orientation had two-peaks distributions. The general insensitivity to correlation changes with the greater (though not perfect) sensitivity to changes in the two-peaks distributions suggests that observers utilized the shapes of feature distributions rather than correlations.

In conclusion, we presented new neurophysiological evidence that numerous spatially irregular, intermixed items can be rapidly parsed into different categories at an early, automatic stage of visual processing. This parsing is driven by global ensemble statistics of feature distributions across the entire visual field. Overall, this finding contributes to our growing understanding of the role of ensemble perception in building relatively rich visual representation beyond the limited-capacity systems^27^.

## Data Availability

The data and Supplemental material can be accessed at: https://osf.io/tymv7/.
